# Matrix Stiffening Enhances DNCB-Induced IL-6 Secretion in Keratinocytes Through Activation of ERK and PI3K/Akt Pathway

**DOI:** 10.3389/fimmu.2021.759992

**Published:** 2021-11-11

**Authors:** Hyewon Chung, Seunghee Oh, Hyun-Woo Shin, Yunam Lee, Hyungsuk Lee, Seung Hyeok Seok

**Affiliations:** ^1^ Macrophages Laboratory, Department of Microbiology and Immunology, Institute of Endemic Disease, College of Medicine, Seoul National University, Seoul, South Korea; ^2^ School of Mechanical Engineering, Yonsei University, Seoul, South Korea; ^3^ Global Technology Center, Samsung Electronics, Co., Ltd., Suwon, South Korea; ^4^ Obstructive Upper airway Research (OUaR) Laboratory, Department of Pharmacology, Seoul National University College of Medicine, Seoul, South Korea; ^5^ Department of Biomedical Sciences, Seoul National University College of Medicine, Seoul, South Korea

**Keywords:** skin, keratinocyte, inflammatory response, IL-6, matrix stiffening

## Abstract

Matrix stiffness, a critical physical property of the cellular environment, is implicated in epidermal homeostasis. In particular, matrix stiffening during the pathological progression of skin diseases appears to contribute to cellular responses of keratinocytes. However, it has not yet elucidated the molecular mechanism underlying matrix-stiffness-mediated signaling in coordination with chemical stimuli during inflammation and its effect on proinflammatory cytokine production. In this study, we demonstrated that keratinocytes adapt to matrix stiffening by increasing cell–matrix adhesion *via* actin cytoskeleton remodeling. Specifically, mechanosensing and signal transduction are coupled with chemical stimuli to regulate cytokine production, and interleukin-6 (IL-6) production is elevated in keratinocytes on stiffer substrates in response to 2,4-dinitrochlorobenzene. We demonstrated that β1 integrin and focal adhesion kinase (FAK) expression were enhanced with increasing stiffness and activation of ERK and the PI3K/Akt pathway was involved in stiffening-mediated IL-6 production. Collectively, our results reveal the critical role of matrix stiffening in modulating the proinflammatory response of keratinocytes, with important clinical implications for skin diseases accompanied by pathological matrix stiffening.

## Introduction

The skin is the interface between the environment and inner tissues and is constantly exposed to diverse external stimuli such as mechanical stresses or toxic chemicals. Keratinocytes, a main component of the epidermis, are known to form physical and immunological barriers against these stimuli through the production of proinflammatory cytokines such as interleukin-6 (IL-6), IL-1α, tumor necrosis factor (TNF)-α, interferon (IFN) γ, and CXC motif ligand 8 (CXCL8) (1–3). These keratinocyte responses are considered to trigger subsequent inflammatory events by recruiting and activating other immune cells in the skin to maintain skin homeostasis (4–6). Thus, keratinocyte-derived proinflammatory cytokines are necessary for understanding the immunological function of the skin barrier and epidermal abnormalities, leading to the pathogenesis of skin diseases

Recently, it has been shown that cells form cell–matrix adhesions within the three-dimensional (3D) extracellular matrix (ECM); thus, external biophysical cues can be mechanically transmitted into intracellular signaling cascades, impacting cell behaviors accordingly (7–9). Keratinocytes, which are mechanoresponsive cells, could adapt to increasing matrix stiffness by altering their proliferation (10), migration (11), differentiation (12), colony formation (13), and epithelial-to-mesenchymal transition (14). To date, the clinical importance of the keratinocyte mechanosensing is underscored by the maintenance of normal skin homeostasis and wound-healing process, in which granulation tissue with locally elevated stiffness is formed (15, 16). However, in addition to normal physiological tissue stiffening as a protective mechanism, pathological tissue stiffening accompanies certain skin diseases, including keloid scar and stiff skin syndrome (SSS) (17). During disease progression, increased matrix stiffness induces a pathogenic immune response. Consistent with this effect, previous studies have demonstrated the enhanced secretion of cytokines such as TNF-α, IL-6, and transforming growth factor-β2 (TGF-β2) in mouse models or patients with these skin-hardening diseases (18, 19). Accordingly, matrix-stiffness-mediated mechanical cues are considered essential for disease pathogenesis *via* the elaboration of keratinocyte-derived cytokines; therefore, targeting the mechanotransduction pathway is clinically highly effective for the treatment of skin diseases with pathological matrix stiffening. Nonetheless, it is not completely understood how matrix-stiffness-mediated mechanical cues regulate inflammatory cytokine production in keratinocytes in coordination with chemical cues during inflammation.

Therefore, in this study, we investigated the effects of altered matrix stiffness on keratinocyte production of the proinflammatory cytokine IL-6, which was highly induced by chemical stimuli in our previous studies (20, 21). We demonstrated that increased matrix stiffness significantly promotes IL-6 production in HaCaT keratinocytes in response to the strong skin sensitizer 2,4-dinitrochlorobenzene (DNCB) but not the non-sensitizer lactic acid. Mechanistically, we found that keratinocytes could sense matrix stiffness through β1 integrin and integrin-mediated focal adhesion kinase (FAK) and activation of ERK and the PI3K/Akt pathway, as possibly downstream molecules of β1 integrin, was involved in DNCB-induced IL-6 production. These findings provide previously unidentified insights into keratinocyte mechanosensing and signal transduction within the context of modulation of skin immune responses *via* cytokine production and its potential impact on pathological skin diseases with matrix stiffening.

## Materials and Methods

### Substrate Preparation

Polydimethylsiloxane (PDMS) substrates (Sylgard 184, Dow Corning Corp., USA) were prepared by mixing the PDMS base with a crosslinker at a ratio of 50:1 to 10:1. PDMS mixtures were degassed under vacuum, spread onto 13 mm diameter glass coverslips or 6-well plates, and cured overnight at 70°C. To functionalize the PDMS substrates with ECM, the surfaces were covered with a solution of 50 mg/mL sulfo-SANPAH (Thermo Fisher Scientific, USA) in water and exposed to 365 nm UV light for 10 min. This process was repeated twice, followed by incubation with 50 μg/mL human plasma fibronectin. The samples were rinsed three times with PBS and sterilized with UVB and 70% ethanol prior to cell seeding. All chemicals were purchased from Sigma-Aldrich (St. Louis, MI) unless otherwise noted.

### Characterization of Mechanical Properties of PDMS Substrate

The mechanical properties of the PDMS substrates were measured using a custom-made indenter consisting of a load cell and an automated stage (22–24). A spherical tip with a diameter of 5 mm was used for the indentation. Young’s modulus of PDMS at various crosslinker concentrations were estimated by fitting an indentation force-depth curve to the Hertz contact model.

### Cell Culture

The human keratinocyte cell line HaCaT was provided by the German Cancer Research Center (Heidelberg, Germany). Normal human epidermal keratinocytes (NHEK) were obtained from Thermo Fisher Scientific (Waltham, MA, USA). HaCaT cells were cultured in Dulbecco’s modified Eagle medium, which was supplemented with 10% heat-inactivated fetal bovine serum (FBS) (Invitrogen, Carlsbad, CA, USA), 1% penicillin–streptomycin (Invitrogen), and 1% non-essential amino acids (Gibco, Thermo Fisher Scientific, USA). NHEK cells were cultured in EpiLife serum-free medium containing EpiLife undefined growth supplement (Thermo Fisher Scientific). The cells were cultured at 37°C in a humidified incubator containing 5% CO2.

### Cell Proliferation Assay

Cell viability was detected using the cell counting-kit-8 assay (Dojindo, Kumamoto, Japan). HaCaT cells were seeded in a 96-well plate with varying elastic moduli (low, medium, and high stiffness) at a density of 1.2 × 10^5^ cells/mL (100 μL total volume/well). At 24, 48, 72, and 96 h after incubation, 10 μL of CCK-8 solution was added to each well, and the cells were incubated for another 4 h at 37°C, in accordance with the manufacturer’s instructions. The OD at 450 nm was measured using a VICTORTM X3 microplate reader (Perkin-Elmer, Waltham, MA, USA).

### Chemical Treatment

Chemicals 2,4-dinitrochlorobenzene (DNCB, Sigma-Aldrich) and lactic acid (Sigma-Aldrich) were dissolved in dimethyl sulfoxide (the maximum concentration of DMSO in the culture medium was 0.1%) and PBS respectively. For *in vitro* treatment, the HaCaT cells cultured on PDMS substrates were treated with 5 μg/mL DNCB or 1 mg/mL lactic acid (Sigma-Aldrich) for 24 h. The culture medium supplemented with 0.1% DMSO or PBS was used as a vehicle control. The concentrations of DNCB and lactic acid used in this study were determined based on prior tests of 75% cell viability (CV75), as described previously (20).

### Western Blotting

Cells were lysed in RIPA buffer containing a protease inhibitor and phosphatase inhibitor (GenDepot, Houston, TX, USA). The protein samples were separated on a 12% SDS-PAGE gel. After electrophoresis, the proteins were transferred to nitrocellulose membranes (Millipore, Bedford, MA, USA), washed with PBS containing 0.05% Tween-20 (PBST), and blocked with PBS containing 5% non-fat dry milk for 1 h at room temperature. The membranes were then incubated overnight at 4°C with primary antibodies against β1 integrin (#4706, 1:1000, Cell Signaling Technology, Danvers, MA, USA), phospho-ERK1/2 (#5726, 1:1000, Cell Signaling Technology), total ERK1/2 (#4695, 1:1000, Cell Signaling Technology), phospho-PI3K (#3087, 1:1000, Cell Signaling Technology), total PI3K (#3087, 1:1000, Cell Signaling Technology), phospho-Akt (#9271, 1:1000, Cell Signaling Technology), total Akt (#9272, 1:1000, Cell Signaling Technology), and β-actin (sc-47778, 1:3000, Santa Cruz Biotechnology, USA). The following day, the membranes were washed three times with PBST and further incubated for 1 h at room temperature with the appropriate HRP-conjugated secondary antibodies (1:5000, Santa Cruz Biotechnology). Proteins were visualized using an enhanced chemiluminescence light-detecting kit (34095, SuperSignal West Femto, Thermo Fisher Scientific).

### Immunofluorescence Cell Staining and Imaging

For immunofluorescence staining, keratinocytes on PDMS substrates were fixed in 4% paraformaldehyde (Electron Microscopy Science) and permeabilized in 0.5% Triton X-100 (Sigma-Aldrich). For actin and nucleus staining, the cells were incubated with Alexa-488-labeled phalloidin (Invitrogen) and Hoechst 33342 (Sigma-Aldrich) in 1% BSA (Sigma-Aldrich) blocking solution. For focal adhesion staining, the cells were incubated with a focal adhesion kinase (Proteintech, Rosemont, IL, USA) primary antibody for 1 h at room temperature. After washing, the cells were incubated for 1 h with Alexa-568-labeled goat anti-rabbit secondary antibody (Thermo Fisher Scientific). All images were obtained using an upright microscope (Nikon) with Plan Fluor 20× (NA 0.5) and S Plan Fluor 40× (NA 0.6) objectives.

### Enzyme-Linked Immunosorbent Assay (ELISA)

After 24 h of incubation under chemical or mechanical stimulation, as described previously (2, 21), ELISA for IL-6 was performed on conditioned media from cells on PDMS substrates, in accordance with the manufacturer’s protocol (R&D Systems, Minneapolis, MN, USA).

### Quantitative Real-Time PCR

For quantitative real-time PCR analysis, total RNA was solubilized in TRIzol reagent (Invitrogen) and extracted according to the manufacturer’s instructions. cDNA was synthesized from 1 μg of total RNA using reverse transcription, and the amount of mRNA was determined using real-time PCR analysis with the SYBR Green qPCR premix (Enzynomics, Daejeon, South Korea) on an ABI real-time PCR 7500 machine (Applied Biosystems, CA, USA). The primer sequences were as follows: human IL-6 forward, 5-AAATTCGGTACATCCTCGAC-3; human IL-6 reverse, 5-CAGGAACTGGATCAGGACTT-3; human β-actin forward, 5-ATTGCCGACAGGATGCAGAA-3; and human β-actin reverse, 5 -GCTGATCCACATCTGCTGGAA-3.

### Statistical Analysis

All statistical analyses were conducted using the GraphPad Prism software (version 8.0) and displayed as the mean ± standard error of the mean (SEM). A *p*-value less than 0.1 was considered to indicate a significant difference (*p < 0.1; **p < 0.01; ***p < 0.001; and ****p < 0.0001).

## Results

### Substrate Stiffness Alters Morphology and Cytoskeletal Organization in Keratinocytes

To investigate the effect of matrix stiffness on keratinocytes, we employed a cell culturing system using PDMS with low (20 kPa), medium (500 kPa), or high (1200 kPa) elastic modulus (**Supplementary Figure S1**). Although there is discrepancy in the references, the elastic modulus of the normal skin is 20~140kPa and the modulus further increases during either normal physiological formation of mature granulation tissue after injury (200kPa~) or pathological excessive ECM production in the skin with stiff keloid scar tissue (100~1000 kPa) (12, 25, 26). Thus, 20 kPa substrate mimicked the stiffness of normal skin, and the substrates with 500 and 1200 kPa were most similar to mature granulation tissues and pathological skin diseases respectively.

First, we cultured HaCaT cells on PDMS substrates with varying elastic moduli for 4 days and observed the influence of matrix stiffness on long-term keratinocyte growth. Cell growth tracking revealed a more rapid increase in the number of cells on the stiff substrate from day 2, and the difference gradually increased over the 4 days, which is consistent with the results of previous studies (10, 12) (**Supplementary Figure S2**).

**Figure 1:**
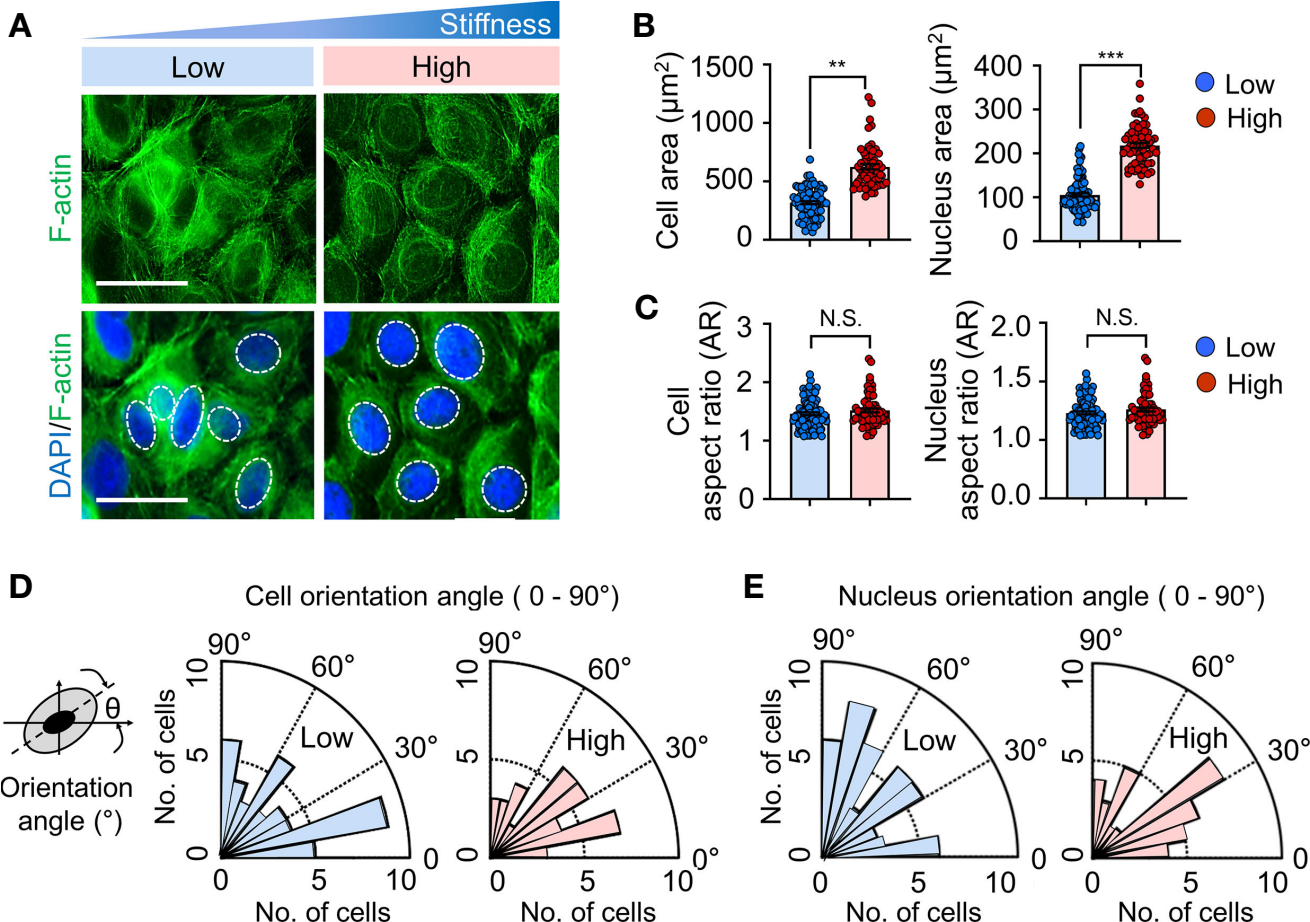
Substrate stiffness alters morphology and cytoskeletal organization in keratinocytes. **(A)** Representative immunofluorescent images showing the nucleus (DAPI, blue) (indicated by the white dotted line) and F-actin (green) in HaCaT cells cultured on substrates with low (20 kPa) and high (1200 kPa) stiffness at day 1. Scale bar = 50 μm. The area **(B)**, aspect ratio [AR, **(C)**], and orientation **(D, E)** of the cell and nucleus were quantified on HaCaT cells cultured on low- and high-stiffness substrates. All images were analyzed using the Cell Profiler software. n = 350-1000 **(B–E)**. Data represent the mean ± S.E.M. **p < 0.01; ***p < 0.001 by Student’s *t*-test. N.S., nonsignificant.

To assess whether the alteration of matrix stiffness causes cytoskeletal remodeling, cell morphology and cytoskeletal organization were evaluated in keratinocytes cultured on substrates with low (20 kPa) or high (1200 kPa) stiffness. Consistent with the results presented in **Supplementary Figure S2**, there were no detectable differences in the number of keratinocytes on day 1; however, the cells cultured on the stiffer substrate (high) were well spread with visible actin stress fibers compared to those on the soft substrate (low) (**Figure 1A**). Additionally, quantification analysis using Cell Profiler (21) showed that the adhesive contact area of cells and nuclei increased dramatically with increasing substrate stiffness (**Figure 1B**). A cell or nucleus was fitted to an ellipse. The aspect ratio (AR) of cell or nucleus was estimated by dividing the long axis by short one of the ellipse (27, 28). There were no significant differences in terms of AR and orientation of cells and nuclei over the range of matrix moduli (**Figures 1C–E**). Collectively, these results indicate that the actin cytoskeleton and nucleus structurally and biochemically adapt to changes in the matrix substrate, which is responsible for transducing diverse mechanical signals into cellular responses.

### Matrix Stiffening Enhances IL-6 Production in Response to DNCB in Keratinocytes

**Figure 2:**
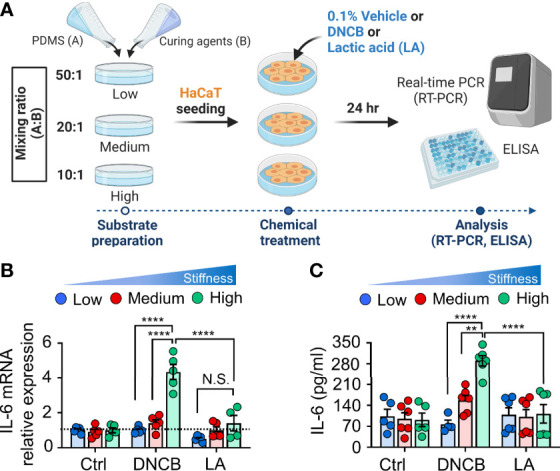
Matrix stiffening enhances IL-6 production in response to DNCB in keratinocytes. **(A)** The experimental design for chemical and mechanical stimulation of proinflammatory response. **(B)** mRNA expression of IL-6 in HaCaT cells cultured on substrates with low (20 kPa), medium (500 kPa) and high (1200 kPa) stiffness in response to 0.1% DMSO (control, Ctrl), DNCB, and lactic acid (LA). Data are normalized to 0.1% DMSO-treated cells (Ctrl) on substrates with low stiffness. **(C)** ELISA for IL-6 in the culture supernatants from HaCaT cells cultured on substrates with low, medium, and high stiffness in response to 0.1% DMSO (Ctrl), DNCB, and LA. n = 4~6/group. Data represent the mean ± S.E.M. **p < 0.01; ****p < 0.0001 by two-way ANOVA. N.S., nonsignificant.

Considering the function of the actin cytoskeleton and nucleus in cellular mechanotransduction to modulate gene expression (29–32), we next investigated whether alteration of the substrate matrix could concurrently influence pro-inflammatory cytokine production. To address this question, we compared the stiffness-mediated production of the proinflammatory cytokine IL-6, which has been shown to be increased in keratinocytes by mechanical stretching coupled with chemical stimulation in our previous study (21) (**Figure 2A**). Without no chemical stimulation, we observed an indistinguishable difference in IL-6 mRNA expression (**Figure 2B**) between keratinocytes cultured on substrates with varying stiffness. However, when DNCB with strong sensitizing potential was added to the cells, IL-6 expression was significantly enhanced with increasing substrate stiffness in keratinocytes. Notably, unlike keratinocytes treated with DNCB, the observed stiffness-mediated IL-6 production was not shown in response to the non-sensitizer lactic acid (LA), suggesting that mechanical cues in concert with strong chemical stimulation are required for IL-6 production. The results showing that increased substrate stiffness stimulated IL-6 in response to DNCB were consistent with those observed at the protein level, as measured using ELISA (**Figure 2C**). A similar higher increase in the level of IL-6 production was found in NHEK cells cultured on the stiffer substrate (**Supplementary Figure S3**). Collectively, we conclude that increased matrix stiffness promotes IL-6 production in keratinocytes upon treatment with chemicals with strong skin sensitizing potential to evoke an inflammatory response.

### Mechanical and Chemical Stimuli Are Coupled to Coordinate FAK Activation

**Figure 3:**
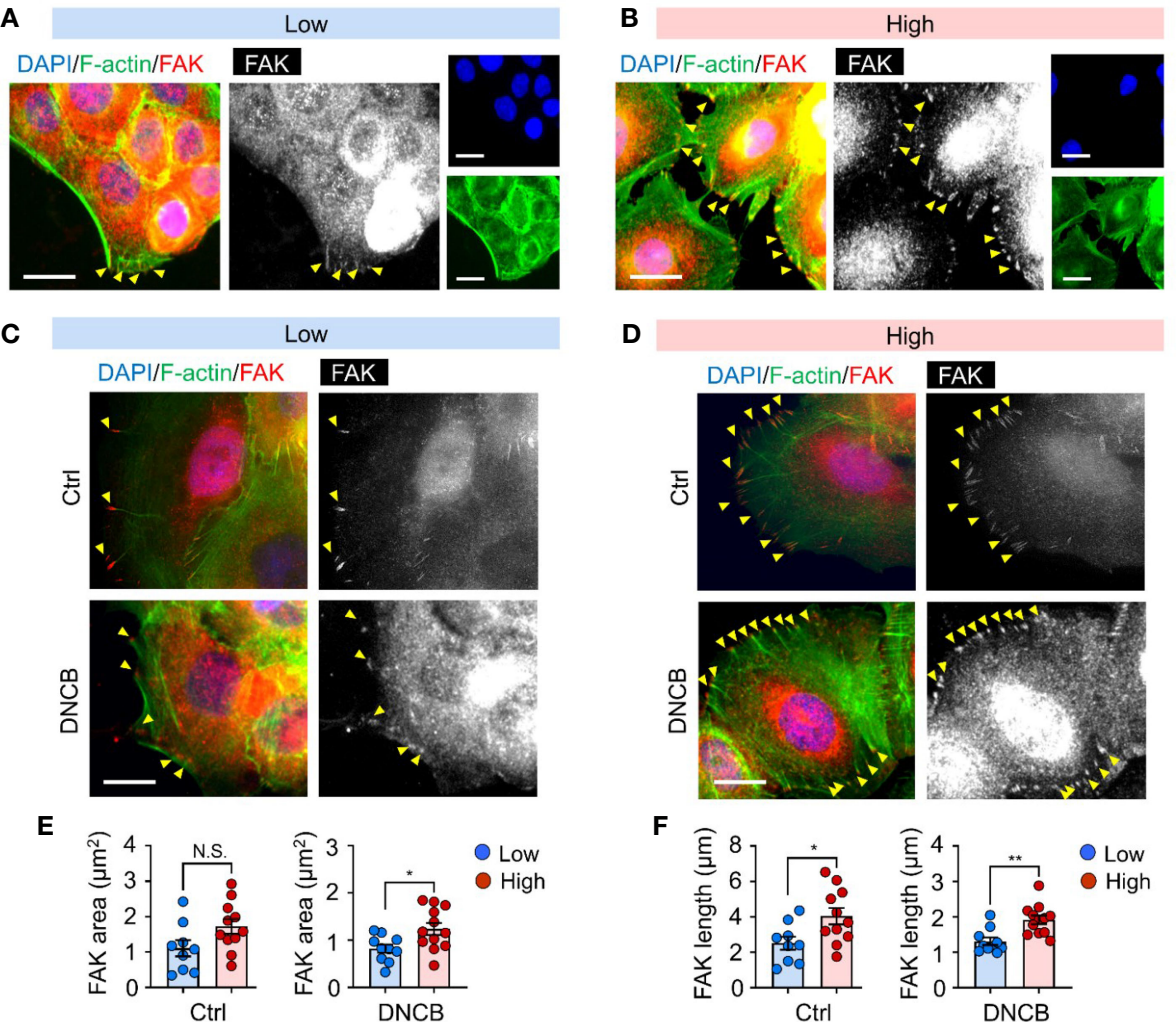
Mechanical and chemical stimuli are coupled to coordinate FAK activation. **(A, B)** Representative immunofluorescent images showing the nucleus (DAPI, blue), F-actin (green), and focal adhesion kinase (FAK, red) in HaCaT cells cultured on substrates with low (20 kPa) **(A)** and high (1200 kPa) stiffness **(B)**. Yellow arrowheads indicate FAK at the cell periphery. **(C, D)** Representative immunofluorescent images showing the nucleus (blue), F-actin (green), and FAK (red) in HaCaT cells on substrates with low **(C)** and high **(D)** stiffness in response to 0.1% DMSO (Ctrl) and DNCB. Scale bar = 25 μm. **(E, F)** Quantification of area **(E)** and length **(F)** of FAK estimated from fluorescent images in **(C, D)**. n = 9~12/group. Data represent the mean ± S.E.M. *p < 0.1; **p < 0.01 by Student’s *t*-test. N.S., nonsignificant.

Focal adhesions play an essential role in integrating mechanical properties of the extracellular matrix into biochemical and transcriptional responses, thereby regulating cell behaviors, including cell adhesion, migration, and differentiation (33, 34). To gain insight into the mechanisms underlying matrix stiffness-mediated IL-6 production in response to DNCB, we first examined the focal adhesion detected by FAK, a central signaling hub between integrin and multiple downstream cellular signaling pathways (35, 36) in keratinocytes cultured on substrates of different stiffness. FAK immunofluorescence imaging revealed that the cells established more adhesive interactions, as evidenced by both the area and length of FAK at the cell periphery, along with more visible stress fibers on the stiffer substrate (high) than on the soft substrate (low) (**Figures 3A, B**). Importantly, the difference in FAK activation between the substrates was more significant after DNCB treatment, further supporting a possible role of FAK in regulating stiffness-mediated IL-6 production (**Figures 3C–F**).

### Matrix Stiffness Modulates IL-6 Production Through Activation of ERK and PI3K/Akt Pathways

Focal adhesion transduces mechanical cues from the altered ECM to regulate cell behavior, in which integrins act as a coupler of mechanotransducers to initiate biochemical signaling (37, 38). In particular, recent studies have suggested that increased matrix-stiffness-dependent β1 integrin expression and clustering promote focal adhesions and subsequent mechanotransduction pathways including FAK-ERK and FAK-PI3K/Akt, which are involved in modulating the pathogenesis of various diseases (38–41). Furthermore, it is notable that β1 integrin-PI3K/Akt signaling pathway is involved in the upregulation of cytokine VEGF in cancer cells (41). Thus, we focused on the β1 integrin and its downstream ERK and PI3K/Akt signaling as possible mechanisms underlying matrix stiffening-mediated IL-6 secretion in keratinocytes.

**Figure 4:**
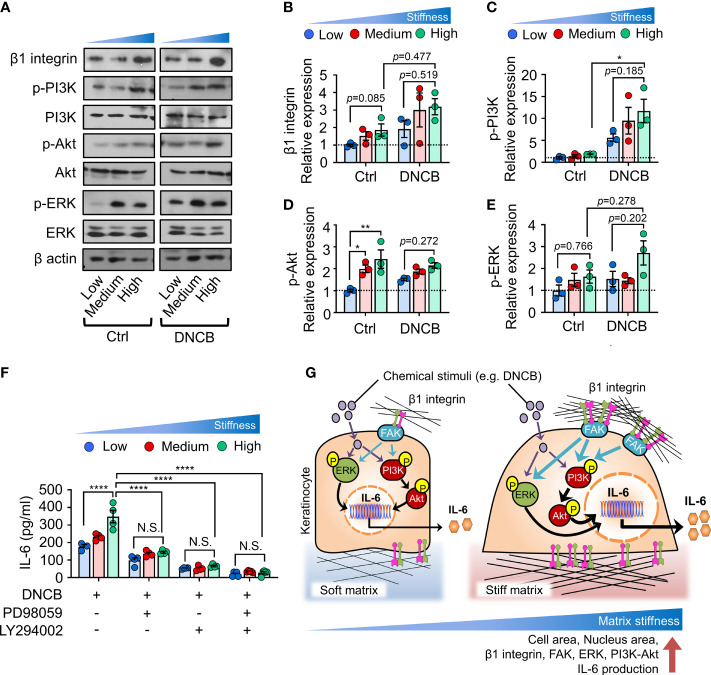
ERK and PI3K/Akt pathways are involved in matrix stiffening-mediated IL-6 production. **(A)** Immunoblot analysis of β1 integrin, p-PI3K, PI3K, p-Akt, Akt, p-ERK, ERK, and β actin in HaCaT cells cultured on substrates with low (20 kPa), medium (500 kPa) and high (1200 kPa) stiffness in response to 0.1% DMSO (Ctrl) and DNCB. **(B–E)** Quantification of the band intensity ratio for β1 integrin:β actin **(B)**, p-PI3K:PI3K **(C)**, p-Akt : Akt **(D)**, and p-ERK : ERK **(E)**. Data are normalized to 0.1% DMSO-treated cells (Ctrl) on substrates with low stiffness **(B–E)**. **(F)** ELISA for IL-6 in the culture supernatants from HaCaT cells cultured on substrates with low, medium, and high stiffness in response to DNCB in the absence or presence of PD98059 or/and LY294002. n = 3~4/group. Data represent the mean ± S.E.M. *p < 0.1; **p < 0.01; ****p < 0.0001 by two-way ANOVA. N.S., nonsignificant. **(G)** A model describing matrix-stiffness-driven regulation of IL-6 production in response to chemical stimuli in keratinocytes. Keratinocytes sense matrix rigidification through the activation of β1 integrin and subsequent FAK, which in turn may lead to phosphorylation of ERK and PI3K/Akt. This mechanotransduction pathway coordinates chemical stimuli such as DNCB to enhance IL-6 production in keratinocytes.

Immunoblot analyses showed that β1 integrin was enhanced with increasing substrate stiffness independent of chemical stimuli (**Figures 4A, B**). Additionally, we observed that matrix stiffening induced phosphorylation of ERK and PI3K, followed by phosphorylation of Akt, as possibly subsequent downstream pathways of β1 integrin activation (**Figures 4C–E**). When DNCB was treated, the difference was more evident. These observations indicate that β1 integrin, ERK and PI3K/Akt pathways, which were activated with increasing matrix, were upregulated in keratinocytes exposed to DNCB.

We next investigated whether blocking ERK and PI3K/Akt pathways prevents matrix stiffness-dependent enhanced production of IL-6. To this end, we inhibited the ERK or PI3K/Akt pathway with PD98059 (ERK inhibitor) or LY294002 (PI3K/Akt inhibitor) and observed an overall decrease in IL-6 production in DNCB-treated keratinocytes (**Figure 4F**). Moreover, upon treatment with PD98059 or LY294002, IL-6 production in keratinocytes on the substrates with the highest stiffness was reduced to levels comparable to those on the least stiff substrate. Co-inhibition of ERK and PI3K/Akt with combination treatment completely abolished matrix-stiffening-enhanced IL-6 production in keratinocytes. Collectively, these results demonstrate that matrix-stiffening-mediated signal transduction through activation of ERK or PI3K/Akt pathway cooperates with chemical stimuli to drive IL-6 production.

## Discussion

Keratinocytes play important roles in the protective machinery of the skin, and experimental evidence suggests that the mechanical properties of the ECM can enable the regulation of keratinocyte behavior and subsequent protective immune response of the skin (10, 42). Thus, further elucidation of the mechanism by which matrix stiffening regulates the inflammatory response of keratinocytes in the context of cytokine production is essential for understanding the role of mechanical cues in the pathogenesis of skin diseases with matrix stiffening and future therapeutic interventions for these diseases.

Here, we showed that increased matrix stiffness promoted proinflammatory cytokine IL-6 production in keratinocytes in response to chemical stimuli. In addition, we uncovered the first detailed molecular mechanism through which keratinocytes integrate chemical and mechanical inputs from the environment to regulate IL-6 production. These findings emphasize biophysical cues, particularly matrix stiffness, as a central regulator of cell signaling in keratinocytes, consistent with previous observations in other cell types (43–45), and highlight the role of the stiffness-mediated mechanotransduction pathway in enhancing proinflammatory cytokine production in response to external stimuli, such as DNCB (**Figure 4G**).

Mechanistically, we found that increase in matrix stiffness enhanced the levels of β1 integrin, FAK and phosphorylation of ERK and PI3K/Akt. The functional link between ERK/PI3K/Akt signaling pathways and IL-6 production was illustrated by the result that stiffness-dependent IL-6 production was abolished through inhibition of either the ERK or PI3K pathway individually or combined inhibition of both. Given the well-described β1 integrin-dependent ERK and PI3K/Akt pathways (38–41) and stiffness-mediated β1 integrin expression observed in this study (**Figure 4**), it is plausible that β1 integrin was involved in IL-6 production. Nonetheless, these data are insufficient to conclude that β1 integrin directly contributes to IL-6 regulation. Furthermore, we could not exclude the possibility that the enhanced several growth factor receptors with increasing matrix stiffness (46, 47) activated ERK/PI3K/Akt pathway. In particular, EGFR activation and downstream phosphorylation of ERK and PI3K have been previously reported in keratinocytes (10). Thus, further studies are required to elucidate the involvement of β1 integrin or EGFR as upstream signaling molecules of ERK and PI3K/Akt pathways and their contribution to the observed stiffness-mediated behaviors in keratinocytes.

This cell–matrix interaction and its effect on cytokine production may be especially significant in light of *in vitro* alternative assays that use keratinocytes as a replacement for animal experiments. Various skin sensitizers stimulate keratinocytes to produce proinflammatory cytokines including IL-6, and these keratinocyte-derived cytokines have been evaluated as biomarkers for discriminating sensitizers from non-sensitizers (20, 48). Several previous studies on *in vitro* irritation and/or sensitization tests using keratinocytes have been based on the culture of cells on plastic surfaces; they have analyzed cytokine production upon chemical treatment. However, plastic culture plates exhibit an elastic modulus of magnitude considerably higher than normal physiological skin tissues and thus could exert excessive forces on keratinocytes. Considering the importance of mechanical cues on IL-6 production, as exemplified in this study, it is suggested that to test the skin sensitizing potential of chemicals under physiological conditions, substrate stiffness should be fully considered.

In summary, our data demonstrate the regulation of IL-6 production in keratinocytes by matrix stiffening and indicate that stiffness-mediated mechanoregulation is a potential contributing factor to the pathogenesis of skin diseases. Consistent with this result, clinical evidence suggests that pathological matrix stiffening is often accompanied by enhanced IL-6 production (19, 49). Therefore, our results could be translated into a novel therapeutic approach for skin-hardening diseases that targets mechanically regulated β1 integrin or ERK/PI3K/Akt pathway, as described in this study, to achieve better outcomes. In addition, a recent study investigating this approach indicated that integrin-targeted therapy prevents skin fibrosis in SSS, supporting the therapeutic potential of the mechano-based approach for the treatment (50).

## Data Availability Statement

The original contributions presented in the study are included in the article/**Supplementary Material**. Further inquiries can be directed to the corresponding authors.

## Author Contributions

HC, SO, HL, and SS designed the research. HC and SO performed the experiments and analyzed the data. HC, SO, YL, H-WS, HL, and SS wrote the manuscript. All authors reviewed the manuscript. All authors contributed to the article and approved the submitted version.

## Funding

This work was supported by the National Research Foundation of Korea (2020R1C1C1012963, 2020R1A4A2002903, 2020R1A2C2010202, 2018R1A2A3075287, 2021R1A4A1032207 and 2021R1A2C2009070).

## Conflict of Interest

Author SO was employed by company Samsung Electronics, Co., Ltd.

The remaining authors declare that the research was conducted in the absence of any commercial or financial relationships that could be construed as a potential conflict of interest.

## Publisher’s Note

All claims expressed in this article are solely those of the authors and do not necessarily represent those of their affiliated organizations, or those of the publisher, the editors and the reviewers. Any product that may be evaluated in this article, or claim that may be made by its manufacturer, is not guaranteed or endorsed by the publisher.
