# Supplementary material for: Matrix Stiffening Enhances DNCB-Induced IL-6 Secretion in Keratinocytes Through Activation of ERK and PI3K/Akt Pathway
Source: Front Immunol. 2021 Nov 11;12:759992. doi: 10.3389/fimmu.2021.759992 (PMC8631934; doi:10.3389/fimmu.2021.759992)
Supplement: Supplementary file 1 [file DataSheet_1.docx]

SUPPLEMENTARY MATERIAL

# Supplement figures

**
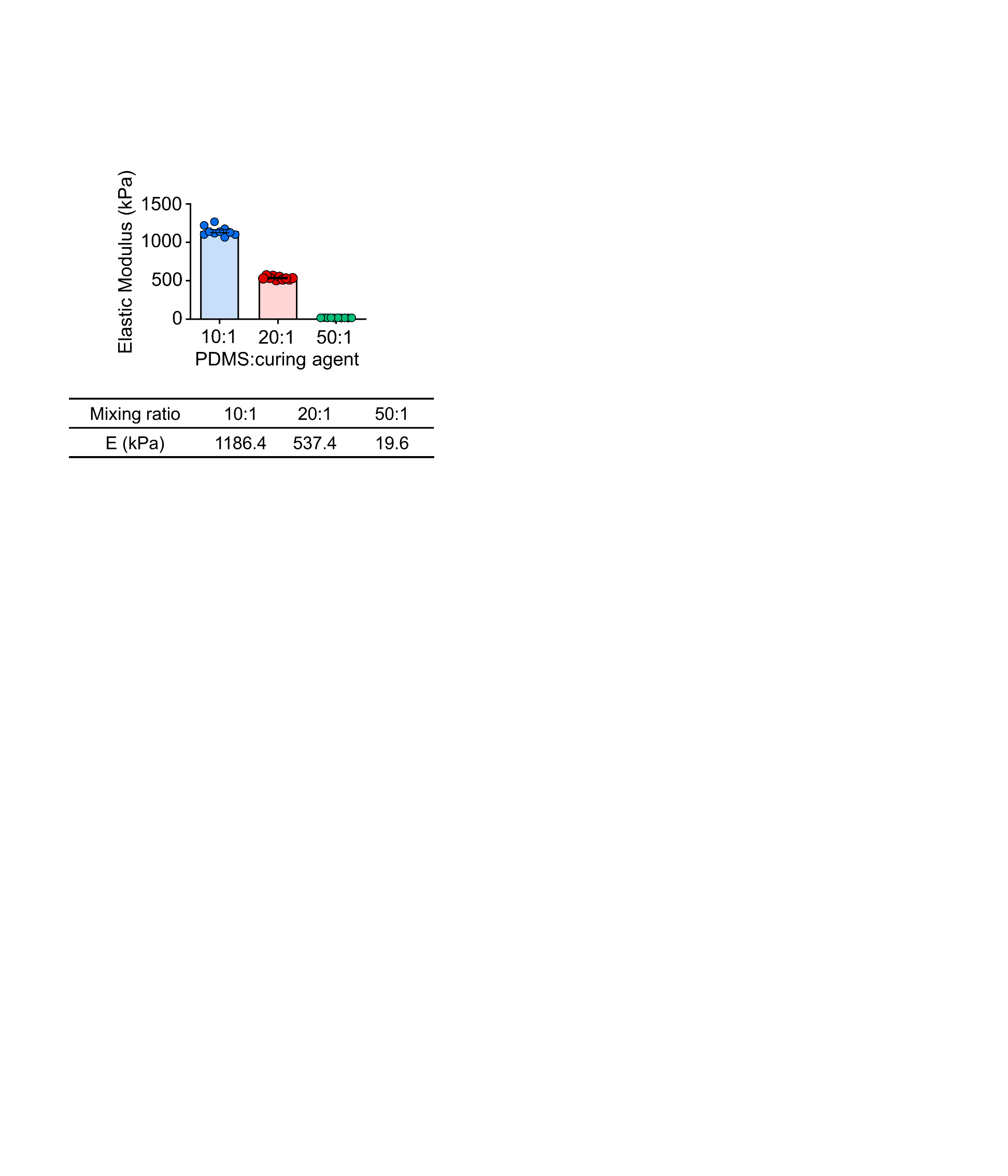
**

**Supplementary Figure 1. PDMS substrate characterization.** Quantification of elastic moduli of PDMS substrates with different mixing ratios, as measured by tensile testing.

**
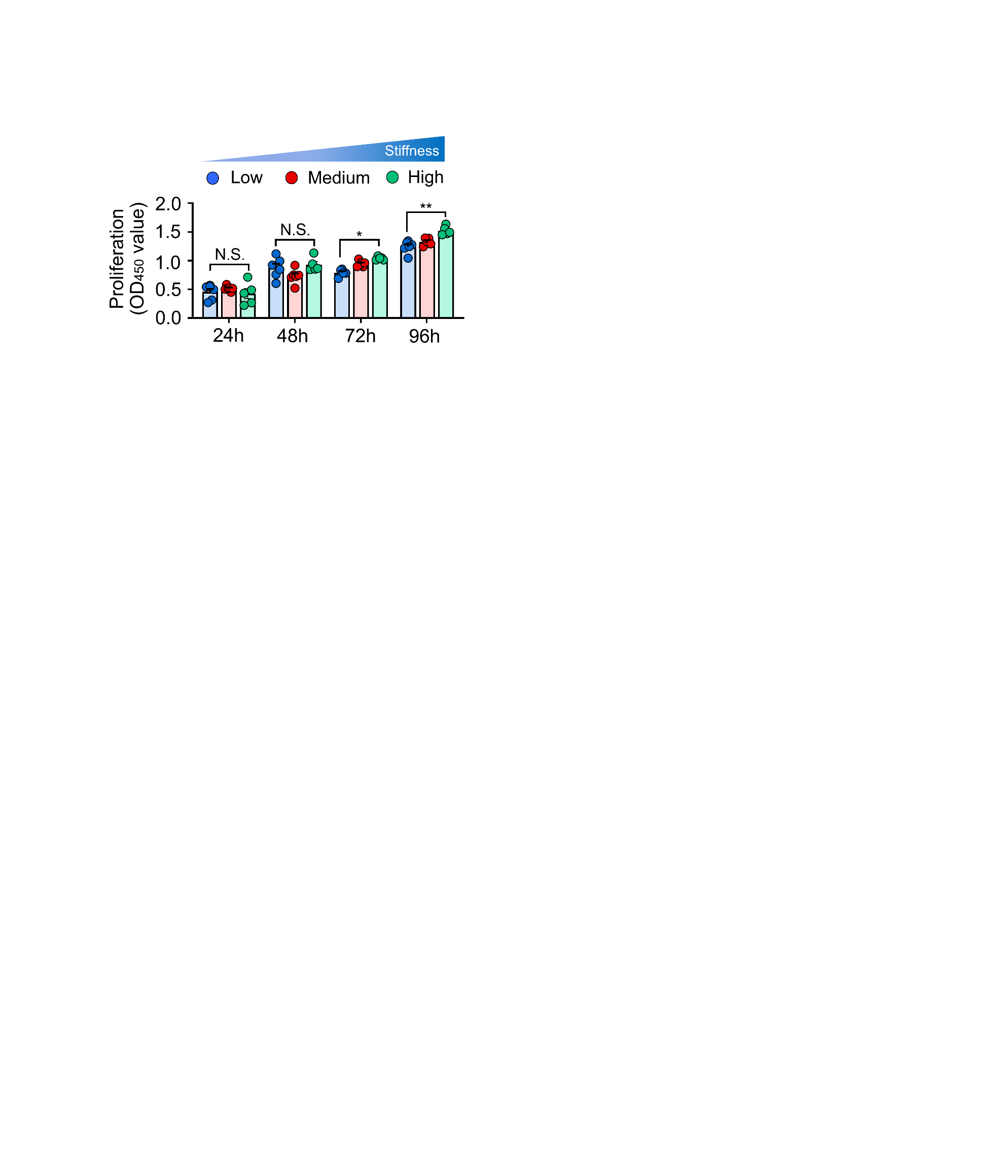
**

**Supplementary Figure 2. Matrix stiffening enhances keratinocyte proliferation.** Proliferation of HaCaT cells cultured on substrates with low (20 kPa), medium (500 kPa) and high (1200 kPa) stiffness at 24, 48, 72, and 96 h after incubation, as assessed using a CCK-8 proliferation assay. n = 5~6/ group. Data represent the mean ± S.E.M. **p* < 0.1; ***p* < 0.01 by two-way ANOVA. N.S., nonsignificant.

**
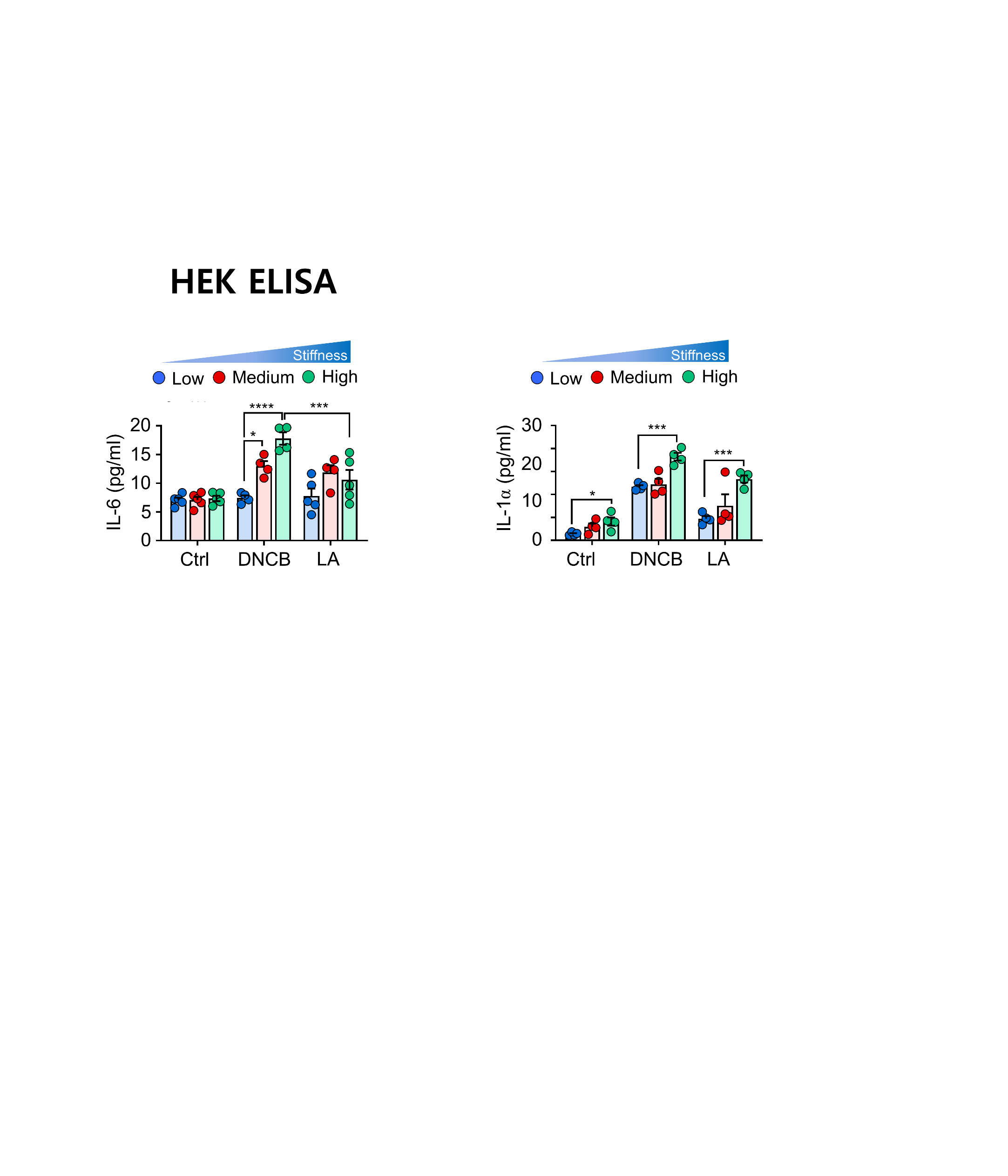
**

**Supplementary Figure 3. Matrix stiffening enhances IL-6 production in response to DNCB in NHEK cells.** ELISA for IL-6 in the culture supernatants from NHEK cells cultured on substrates with low, medium, and high stiffness in response to 0.1 % DMSO (Ctrl), DNCB, and LA. n = 4~5/group. Data represent the mean ± S.E.M. **p* < 0.1; ****p* < 0.001; *****p* < 0.0001 by two-way ANOVA.
